# Clinical Society of Maryland

**Published:** 1892-04

**Authors:** 


### CLINICAL SOCIETY OF MARYLAND.

Baltimore, February 5, 1892.

The 261st regular meeting of the society was called to order by the president, Dr. Robert W. Johnson.

Dr. W. B. Platt read a paper on "Free Dispensaries, or the Physician and the Poor." Dr. Platt, in his dispensary work, adopts, as nearly as possible, the following plan: Inhabitants of certain squalid alleys, well known to him, are treated without question. The destitute and forlorn, whose aspect is unmistakable to one having dealings with the poor, come in first of all for treatment. Mechanics, artisans or laborers, out of work and out of money, and the poor families of drunken and worthless men, are all entitled to free treatment. Adults who have to pay for their board and lodging out of wages less than $5 per week, are treated free. House servants, earning $10 and $12 per month, can and do pay physicians for advice.

Dr. I. E. Atkinson said: This subject, as Dr. Platt has pointed out, bears upon the patients, the physician in attendance and the profession at large. The abuses of dispensaries is a world-wide complaint, and the difficulties that stand in the way of correcting them are almost insuperable. In the first place, the presence of a person at the dispensary is a confession of poverty, and when questioned in regard to their financial condition, nearly every patient is prepared to say that he is unable to pay the fees of a physician. Occasionally one encounters patients who, when questioned, avow their ability to pay, and are properly excluded.

I think that the evils of dispensary service are more apt to be developed in dispensaries other than those in which patients are used for clinical purposes. The presentation before a class of students is, to persons who are not degraded, a very disagreeable procedure, and they will refuse to come again unless compelled by necessity.

What kind of patients are entitled to relief? Every one admits that the pauper is a proper person. There is not so much unanimity of opinion with regard to the relief of those persons who are brought to that condition by their own vices. Never mind what his faults nor what his vices, nor how utterly beyond the pale of ordinary sympathy he is, as soon as he is sick he becomes a worthy object of charity. In this way medical charity differs from almost every other kind of charity.

Dr. Platt mentions another class that especially appeals to my sympathy, viz.: the wage-earner who makes $10 per month. As to whether or not he shall pay, depends entirely upon how much he is called upon to pay. A fee of $1.00 would be 10 per cent. of his income for the month, and his medicine would perhaps cost 5 per cent. more. It may be that he should not be the beneficiary of a free dispensary, but of a provident dispensary—the absence of which, in Baltimore, I very much regret. I further believe that the man who earns $1.00 or $1.50 per day and supports his family is entitled to a modified relief. This man, by careful economy, is able to keep his family alive, but he cannot support them in comfort. Just as soon as a member of his family falls sick, his expenditures are enormously increased, while his income remains the same, or is diminished. If he himself falls sick, the income stops, while expenses increase. I think that one of the great needs is that modified form of charity, which we recognize as a provident dispensary. This idea of a provident dispensary is not a new one. The individual pays into it so much per month, and his membership entitles him to receive the services of good, intelligent physicians, who are properly paid for their services by the association, and gets his medicines at a reduced rate. Membership in the dispensary is only granted to those who receive a certain maximum of wages. Such dispensaries have been in existence in England for fifty years, yet the number is small. The justice of them, the propriety of them and the benefits to be derived from them are so manifest that it is difficult to understand why it is that such a limited popularity should be accorded to them.

That there is dreadful abuse in dispensary practice I am convinced; but that the abuse is not altogether on the part of the patients, I am also convinced. There are few ordinary day laborers who feel able to pay the full fees of physicians and the prices of the pharmacist. Some do it from pride, some from principle, and some they know not why. But in case of continued sickness it is absolutely impossible for them to pay physicians' fees, and they are forced into increasing debts which they know they cannot pay. I am an advocate of that form of relief which shall not pauperize the individual, but will enable him to secure for himself and family proper professional advice and necessary medicines without too great a strain on his purse.

DR. PLATT: I think Dr. Atkinson's point in regard to there being less abuse in dispensaries where patients are used for clinical material is well taken; and yet the great howl that has gone up recently has been on account of a dispensary which is used almost exclusively for purposes of instruction. I think there are many persons who are perfectly shameless about getting charity. There is generally a look about a person who lives poorly and miserably that enables you to spot them as quickly as you can tell a wharf rat from a common one. They have poverty written all over them. There is a middle class, whose earnings are not much, yet who have deposits in the savings bank, and ought to pay. There are physicians who would make a reasonable number of visits at half price, and they can get reduced rates at the pharmacists. As to having patients pay at a dispensary, that has been tried. The only thing that has not been tried thoroughly is to carefully investigate each patient by a visit to his home. I have had people come to me at the dispensary who owned houses and had bank accounts ; others with a large number of children, all receiving good salaries.

I think the key to the whole matter is to look up each individual, and see whether or not he can pay. I think there are very few physicians in this room who charge all persons alike. If a patient cannot pay my full fee, I treat him for less.

DR. HERBERT HARLAN : I have had experience with different dispensaries ever since my student days. I believe that at the dispensary of the Maryland University, where patients are used for clinical purposes, there is very little imposition. It may be on account of the large class of students, for the tendency of people is not to go before a class of students. I have known a good many patients to go to that dispensary on other days of the week, and to absent themselves on the days of the clinic. There is, however, quite a large class of people who like to hear their cases discussed. The Baltimore General Dispensary is not imposed on much, because the physicians visit the patients' houses and see whether they can pay or not. The great abuse is undoubtedly in the special dispensaries. We have tried a good many devices to prevent those who ought to pay from receiving services free. One was for the physician to question them as to their ability to pay. Sometimes they answer yes, sometimes no. Some say they can pay, but others who can pay are treated free. Here is the point that I want especially to raise here : At a special dispensary it is a daily occurrence for patients to say, "Dr. So and So, my family physician, sent me here to have my case treated." Physicians themselves are not as particular about these things as they might be. We ask such people if they pay their family physicians, and they reply, "Certainly we do." Then we refuse to treat them. We have tried in another way to prevent abuse, viz.: by having a clergyman, who is regularly employed for the purpose, to go about the waiting-room and question the patients, and act as judge as to who shall or shall not be treated. This, I think, is a move in the right direction. We are indebted to Dr. Platt for calling our attention to this matter, and we ought all to make an effort to do away with the abuses.

DR I. E. ATKINSON: The physician who charges but small fees knows that in many cases his patient cannot pay the fees of a special practitioner. I frequently have had patients who pay me go to a special dispensary. They do not ask my opinion about it. They say they cannot pay specialists' fees. I think the standard in regard to this class of patients should be a little different from that of the class going to the general dispensaries.

DR. J. EDWIN MICHAEL read "A Report of Eight Additional Cases of External Perineal Urethrotomy without a Guide"—these cases being in addition to nine cases already reported by him in the spring of 1887.

DR. PLATT thought that, considering the difficult nature of the operation, the success of Dr. Michael was astonishing and very unusual.

DR. ROBERT W. JOHNSON spoke on "A Convenient and Comprehensive Method of Instrument Disinfection," and exhibited the apparatus which he devised and used. Dr. Johnson boils everything except himself, his patient, and the rubber tissue. He boils ligatures, instruments, needles, gauze, etc., and also the trays which hold them. The boiler is a plain tin one, large enough to accommodate the trays, with spigot attached near the bottom. A nest of elongated trays of granite ware is found most convenient. Before leaving his office, he goes over the instruments that will be required and puts them in a tray. The dressings to be used are put in another tray, and so on and finally the trays are built up one upon another, and all are put into the boiler, which is put in the back of the wagon. At the patient's house the boiler is filled up with boiling water, put upon the stove and boiled for twenty to thirty minutes, while the patient is being prepared for operation. When ready for operation, the trays are lifted out by means of sterilized button-hooks. The boiler is put in an elevated position, a rubber tube attached to the spigot, and the boiled water is used for irrigation. It makes no difference whether knives or dressings touch the sides of the trays, for they are quite aseptic.

DR. HERBERT HARLAN asked what means were taken to prevent the rusting of instruments in boiling. He had noticed the curious phenomenon that the steel blades of a set of knives with aluminum handles rusted more readily than those of knives with ivory handles.

DR. CHUNN asked Dr. Johnson's method of preparing his hands for operation.

DR. JOHNSON: By adding a slight amount of bicarbonate of soda to the water, rusting of instruments during boiling is prevented. I sometimes use bichloride on my hands, and sometimes potassium permanganate, cleaning it off with oxalic acid. The latter is probably the best method.

